# Updated Guidelines for the Orthodontic Management of Traumatized and Endodontically Treated Teeth: A Review Study

**DOI:** 10.7759/cureus.28943

**Published:** 2022-09-08

**Authors:** Asma Bakkari, Fahad Bin Salamah

**Affiliations:** 1 Orthodontics and Dentofacial Orthopaedics, King Saud Medical City, Riyadh, SAU

**Keywords:** risk factors, root resorption, root canal treated teeth, dental trauma, orthodontic considerations, orthodontic management, guidelines

## Abstract

Traumatic dental injuries (TDIs) are injuries affecting the teeth, periodontium, and surrounding soft tissues. A significant percentage of candidates for orthodontic treatment suffer from previous TDIs to their permanent incisors that mostly remained untreated. Orthodontic treatment of such teeth might be associated with an increased risk of further pulpal and periodontal consequences, especially in teeth with a previous onset of root resorption that has occurred following the trauma. Orthodontic treatment planning can also be challenging for previously endodontically treated teeth. Clinicians should be aware of the techniques and the appropriate time to proceed with orthodontic tooth movement of traumatized and endodontically treated teeth, whether it was secondary to deep carious lesions or TDIs, and about the risks involved.

This review was done in order to provide an evidence-based approach regarding the orthodontic management of traumatized and endodontically treated teeth and the current recommendations for orthodontic tooth movement of such teeth.

## Introduction and background

Traumatic dental injuries (TDIs) are injuries that affect the teeth, periodontium, and surrounding soft tissues. They have a high prevalence, comprising 5% of all traumatic injuries in people seeking first aid and up to 17% of all bodily injuries among preschool children [1]. Children and teenagers, who comprise the majority of orthodontic candidates, are the population mostly affected by TDIs [2].

A significant percentage of candidates for orthodontic treatment suffer from previous TDIs to their permanent incisors that mostly remained untreated [3,4]. A high prevalence of 10.8% has been reported for orthodontic candidates with previous TDIs before the onset of orthodontic treatment [3]. Orthodontic treatment of such teeth might be associated with an increased risk of further pulpal and periodontal consequences, especially in teeth with a previous onset of root resorption following the trauma [5]. Orthodontic treatment planning can also be challenging for previously endodontically treated teeth, and hence, clinicians should have knowledge of the techniques and the appropriate time to proceed with orthodontic tooth movement of traumatized and endodontically treated teeth, whether it was secondary to deep carious lesions or TDIs, and about the risks involved [5,6]. This review was developed to provide an evidence-based approach regarding the orthodontic management of traumatized and root canal-treated teeth and the current recommendations for orthodontic tooth movement of such teeth.

## Review

Effect of orthodontic tooth movement on traumatized teeth

Pulp Vitality

Evidence was found to be insufficient and controversial regarding the development of pulp necrosis following orthodontic movement of teeth with previous TDIs [5,6]. One retrospective study has found a higher prevalence of pulp vitality loss in teeth with previous TDIs undergoing orthodontic treatment. However, the study numbers are too small and inconclusive [3,4]. Another review established that orthodontic tooth movement may develop blood flow disturbances in the pulp, vacuolization and, rarely, pulp necrosis [7]. It has also been reported that maxillary incisors with severe previous TDIs have a higher risk of pulp vitality loss with orthodontic extrusion [8]. In summary, it is not possible to say whether orthodontic movement of teeth with TDIs will increase the risk of vitality loss above that of uninjured teeth; however, all orthodontists must be aware of these risks and the possible need to undertake pulp vitality tests whenever necessary [5,3].

Root Resorption

When the protective pre-dentin or pre-cementum gets removed or altered after an injury, it results in inflammation of the pulp or periodontium, which in turn induces root resorption with multinucleated clastic cells similar to those seen in bone resorption [9]. Despite the fact that this condition is mainly asymptomatic and frequently overlooked in diagnosis, it could lead to tooth mobility and even tooth loss if not diagnosed early and treated properly [10,11]. When 1-2 mm or one-fourth of the root length is lost, root resorption is considered clinically significant. However, permanent coronal mobility is only expected when the root is less than 9 mm in length [6]. Severe root resorption, when more than one-fourth of the root length or >5 mm resorb, occurs in 1%-5% of patients during orthodontic treatment [7]. Luxation injuries are the most likely injuries that may lead to root resorption [9]. There are many types of root resorption; however, this review only focus on three main types as they are the most relevant to the topic of discussion [3].

External surface resorption: This is a sub-clinical self-limiting process that occurs as a result of a small localized and limited injury to the root cementum or surrounding periodontium. Without a constant stimulus, the osteoclastic and resorptive activity arrests after two to three weeks from the time of the injury and cemental healing occurs, where the neighboring intact cementoblasts repair the defect, along with periodontal ligament reattachment [12]. Substantial evidence has established that orthodontic tooth movement can result in external surface resorption [3].

External inflammatory root resorption: This process occurs in the presence of an untreated infection of the root canal system combined with mechanical damage to the cementum during the trauma, or if the cementum has been lost as a result of external surface resorption [10,13]. The main aim while managing external inﬂammatory resorption is to destroy the bacteria and eliminate the infection. This can be achieved by performing excellent root canal treatment with particular emphasis on the use of speciﬁc intracanal medicaments [10].

Replacement resorption: This is the process of the replacement of the root surface with bone, which is also known as ankylosis [12]. This occurs after a severe traumatic impact, particularly after avulsion and intrusion, affects the periodontal membrane in the absence of infection and bacterial stimulus within the root canal system. With time, the entire root might get replaced by bone [3,13,14]. Ankylosis can be detected 2-12 months after the injury, and it is characterized by a high metallic tone upon percussion, infra-occlusion, and obliteration of the periodontal space in the radiograph [6].

Risk factors for root resorption

Risk factors associated with an increased incidence and severity of root resorption include root morphology, length of orthodontic treatment, orthodontic force type and magnitude, and previous dental trauma [3,15].

Root Morphology

The literature established that morphologically abnormal roots are more vulnerable to root resorption while normal and blunt tooth roots are found to resorb the least. Pipette-shape root forms are the most susceptible for root resorption [7,16]. It has been recommended to obtain radiographs three months following the onset of orthodontic treatment in teeth with pipette-shaped or blunt apices [3]. Data is conflicted regarding the initial length of teeth roots and the degree of associated root resorption. It has been found that small roots have a greater risk of root resorption than average length roots and the amount of resorption is almost twice more than with other root forms. However, some believe that longer roots are more susceptible to resorption because they tend to displace farther for equal torque [7]. It has also been established that normal and wide root forms of central incisors are preventive factors and have a decreasing risk of root resorption, while teeth with narrower roots have a slightly increased risk for root resorption [7].

Length of Orthodontic Treatment

The length of orthodontic treatment is considered an important factor that may cause root resorption. Many studies have shown that the severity of root resorption is related to the duration of orthodontic treatment. The amount of root resorption during orthodontic treatment is known to be 0.9 mm per year [7,17]. Other studies proposed that root resorption may commence in the early stage of orthodontic treatment, particularly for teeth with long, narrow and deviated roots [7,18]. A study that investigated root resorption revealed that although light orthodontic forces did not exhibit a remarkable root resorption difference between four and eight weeks of buccal force application, the extent of resorption markedly increased from 8 to 12 weeks of force application. Thus, orthodontic force application for a longer duration can increase the risk for root resorption even when the forces used are as light as 25 g [11]. Several contemporary studies found no relation between root resorption and the length of orthodontic treatment [7,11].

Force Application Method

The literature agrees that jiggling movement and movement caused by using intermaxillary elastics likely increase the risk of root resorption [19]. Intermittent forces are considered to be a safer method to prevent signiﬁcant root resorption, although not very practical when compared to continuous forces, considering how the intermittent force prevents the development of hyalinized areas, or the way it allows for the restoration of blood circulation by reorganization of the hyalinized periodontal ligaments when forces are not active [11,20]. Continuous forces provide no time for periodontal tissues and blood vessels to repair, and therefore, may lead to a higher level of root resorption [7,20]. A study found that when children with class II malocclusion were treated with a bionator followed by ﬁxed appliances (two-phase), they experienced fewer incidences of moderate/severe root resorption. On the other hand, when children with the same malocclusion were treated with ﬁxed appliances alone (single-phase), they had the most resorption. Nevertheless, the difference between the two groups was not statistically signiﬁcant [11,21].

Type of Orthodontic Tooth Movement

Root resorption was established most often after orthodontic intrusion with four times more incidence than that observed with orthodontic extrusion [7,11]. The orthodontic rotation of teeth, especially in single-root teeth, resulted only in minor injuries of periodontal tissues. Bodily movement has been claimed to induce less risk of root resorption than tooth tipping, owing to the fact that force distribution along the root length during bodily movement is less than that of tipping movement where the forces concentrate at the root apex. However, other researchers state that tooth tipping induces less root resorption than bodily movement [7,17].

Force Magnitude

Heavy orthodontic force was found to cause notably more root resorptions than the light orthodontic force, as high force levels tend to cause extensive damage in the periodontal ligament [19,11]. A force magnitude of 20-26 g/cm^2^ or higher can lead to ischemia in the periodontal tissues, which may further cause root resorption. Root resorption stops when orthodontic forces decrease to less than 20-26 g/cm^2^ [7,17,20]. Optimal orthodontic force magnitude that does not cause root resorption has to be 7-26 g/cm^2^ on the root surface area [22]. Some studies found no difference in root resorption at high and low orthodontic force levels (50-200 g); however, it is still not recommended to exert high force levels on the teeth [19].

Dental Trauma

Dental trauma may cause root resorption by itself without orthodontic treatment. Teeth with previous TDIs and root resorption are prone to further loss of root structure when orthodontically moved. Investigations on the effect of previous TDIs on root resorption during orthodontic treatment are conﬂicting and few in number [3,7]. One study revealed that the average resorption was 1.07 mm for teeth with TDIs that went through orthodontic movement, compared to 0.64-mm resorption for sound teeth [23]. It has also been established that when teeth exhibit minor resorption after six to nine months of orthodontic treatment, further tooth movement signiﬁcantly increases the risk of severe root resorption [3].

Endodontic Treatment

There are conflicting data in the literature on the risk of root resorption in endodontically treated teeth during orthodontic treatment. This may be due to differences in the etiology of pulp necrosis [6]. It has been established that as long as there is normal root morphology, there is no signiﬁcant difference between endodontically treated teeth and vital teeth in the root resorption when same orthodontic forces are applied [3,6].

Assessment and diagnosis

Obtaining History

It is essential to take a comprehensive dental history for all new orthodontic patients, with special attention to the history of dental trauma incidence and the treatment provided, as well as the history and the initial diagnosis of endodontically treated teeth. Even in the absence of history of a TDI, it is essential to perform comprehensive clinical and radiographic assessment as patients may not always recall a trauma incident [3,5,15].

Clinical Assessment

The assessment of color is necessary as any changes in the crown color can provide some insight regarding the pulp status of traumatized teeth over time. These changes can be a result of pulp necrosis, pulp tissues with calcific metamorphosis, or they might be an indication of developing an internal or external resorptive process [24]. An examination for sinus tract, swelling, mobility, and tenderness over the tooth apex should be done [3]. The response to percussion test is also important as a high metallic sound often indicates ankylosis, while a duller sound may indicate a root fracture. A sensibility test should be performed whenever necessary. The temporary loss of sensation is a frequent finding during post-traumatic pulp healing, especially after luxation injuries. Therefore, the negative response to pulp sensibility testing is not conclusive for pulp necrosis in traumatized teeth. Moreover, sensibility tests should never be investigated separately from other clinical and radiographic findings [2,3]. It is preferable to use multiple tests, such as thermal tests and electric pulp testing (EPT), as they are both subjective and therefore have compromised accuracy [5]. Teeth opposite or adjacent to the traumatized teeth should always be examined as they may have also been affected at the time of the dental trauma [3,5,6].

Radiographic Assessment

A baseline, pre-treatment and routine radiographic update every six to nine months into the treatment is generally recommended, with such frequency decreased to three months depending on the type of orthodontic treatment provided, the severity of the TDI, and the anticipated complications [3,5]. Standardized long cone periapical radiographs are the first choice for the diagnosis of dental trauma. For the detection of root fractures, preferably two radiographs should be taken at different angulations. Root growth continuation is considered the most conclusive clinical sign of recovered pulpal vitality in immature teeth following a TDI. Therefore, it is advisable to include both the injured tooth and its uninjured counterpart when taking periapical radiographs of traumatized teeth for direct comparison [3]. Cone beam computed tomography (CBCT) is indicated in some cases to identify areas of root resorption not identifiable on a conventional radiograph, and when the degree of root resorption is uncertain [5].

General considerations for the orthodontic management of traumatized and endodontically treated teeth

Informed consent must be obtained from the patient or the patient's guardian, and they must be informed about the risks involved during orthodontic treatment of teeth with a history of TDIs, such as root resorption and pulp necrosis, with these risks speciﬁcally outlined and stated in the informed consent form [5,15,19].

A baseline and regular radiographic review must be done as mentioned earlier. A review suggests taking periapical radiographs of the teeth involved after six months of treatment [19]. When minimal root resorption is observed in the periapical radiograph after six months, treatment should be paused for two to three months with passive arch-wires. When the resorption is sever, however, reassessment of the treatment objective is recommended and alternative treatment options should be discussed and implemented [19]. For traumatized teeth with an immature apex, one should wait for radiographic evidence of continued root development. Clinical and radiographic follow-ups should be performed at six months, one year, and two years [3].

Alternative treatment options in the case of severe resorption include using prosthodontic solutions to close spaces, or leaving the affected teeth out of active arch-wires, or performing inter-proximal stripping (IPR) instead of extractions, or early ﬁxation of resorbed teeth. Orthognathic surgery may also be appraised in severe resorption cases; however, it may not be reliable to prevent further root resorption [19].

If evidence of pulp necrosis and bacterial infection was detected, endodontic treatment is required prior to orthodontic treatment [6]. Moreover, immediate commencement of root canal treatment is recommended after severe TDIs where external inﬂammatory resorption is likely to occur [10]. The use of a corticosteroid/antibiotic paste (e.g., Ledermix paste) intracanal medicament was found to be particularly useful in the prevention and management of external inﬂammatory resorption [10].

The modification of the arch-wire sequence to reduce the orthodontic forces is strongly advised when movement of traumatized teeth is considered. It is rational to utilize forces as light as possible with longer intervals between activations [5,19]. Thermal nickel-titanium arch-wires are recommended for aligning recently traumatized teeth. They demonstrate remarkably lower force-deflection curves than superelastic arch-wires. During space closure, it is advised to limit the torquing force on the traumatized teeth by using an arch-wire with reduced dimensions (e.g., 0.016-/0.022-inch stainless-steel wire) [5]. It is unfavorable to implement the new technique of using the light-force rectangular wires as initial wires for teeth with a history of TDIs. By using them, the jiggling movements during the initial stage of treatment is increased, which may expose the root to further resorption [19].

Evidence suggests the priority of using self-ligating (SL) brackets for patients with more vulnerable maxillary central incisors or reduced crown-root ratio who are receiving orthodontic treatment. In SL brackets, arch-wires could have more free space in slots than in non-SL brackets, owing to the rubber elastics and steel ligatures. This could result in lower frictional force in the SL slot and smaller force to the vulnerable teeth in the initial alignment [25]. However, more clinical trials are required to provide more reliable evidence [26]. Clear aligners have been found to have less prevalence and severity of apical root resorption when measured on CBCT, compared to fixed appliances [27].

Teeth with unfavorable prognosis should be assessed by a specialized interdisciplinary team where treatment modalities such as premolar transplantation, decoronation, dental implant, or fixed partial denture could be considered [3,5].

Orthodontic management of specific TDIs

TDIs can be categorized into the following different groups, and each group calls for specific orthodontic management: minor damage to the periodontium, moderate/severe damage to the periodontium, crown and crown/root fractures, root fracture, endodontically treated teeth secondary to trauma, and endodontic challenges.

Minor damage to the periodontium

The orthodontic outcome for these teeth is similar to that for non-traumatized teeth (especially those with open apices), because the damage risk to the periodontal ligament is minimal. Injuries with minor damage to the periodontium include concussion and subluxation.

Concussion

Definition: Concussion is an injury to the tooth periodontal structure without mobility or displacement. The tooth is usually tender to percussion with no other symptoms [5,28].

Orthodontic management: An observation period of three months with a radiographic review is recommended to check for root resorption and periapical pathology prior to orthodontic tooth movement commencement [3,5,6].

Subluxation

Definition: Subluxation is an injury to the tooth periodontal structure that results in abnormal mobility. The tooth exhibits no displacement and is tender to percussion, with sulcular bleeding from the gingiva [5,28].

Orthodontic management: An observation period of three months with a radiographic review is recommended to check for root resorption and periapical pathology prior to orthodontic tooth movement commencement [3,5,6,28].

Moderate/severe damage to the periodontium

When a moderately or severely traumatized tooth is undergoing orthodontic tooth movement, it is expected to have a poorer prognosis, especially if inflammatory or replacement root resorption occurs [6]. This applies for extrusive luxation, intrusive luxation, lateral luxation, and avulsion traumas.

Extrusive Luxation

Definition: The tooth appears longer than the adjacent due to outward displacement and will exhibit mobility. A very high incidence of loss of tooth vitality has been reported after this type of TDI [5,28].

Orthodontic management: An observation period of 6-12 months before orthodontic treatment is recommended in order to ensure that periodontal healing is complete and to avoid extra-inflammatory stimuli, which may further damage the protective cementum layer and increase the risk of ankylosis. Another review suggests that at least three- to six-month observation period should pass before the commencement of orthodontic movement [5,6,28]. If there was a delay in repositioning the traumatized teeth following the trauma, orthodontic intrusion of these teeth might cause complications, such as vitality loss, and reduced crown-to-root ratio and bone support [28]. If extrusion was minimal, a three-month observation period is recommended [3].

Lateral Luxation

Definition: This is when the tooth is displaced in a palatal or lingual direction. The tooth exhibits no mobility and may give a high metallic sound to percussion [5].

Orthodontic management: When there is interference with occlusion, opening the bite becomes mandatory. One should utilize bite-raising techniques such as glass ionomer (GIC) stops or using a removable appliance with bite planes for such cases. Adjunctive orthodontic treatment aims to remove the premature contact only rather than definitively repositioning the tooth [5]. Flexible wires (e.g., 0.012- or 0.014-inch NiTi) are typically used to reposition the tooth, as they deliver a low force and large range during an extended period of time [29]. An observation period of 6-12 months before comprehensive orthodontic treatment is recommended for complete periodontal healing [5]. If lateral luxation was minimal, an observation period of three months is recommended [3].

Intrusive Luxation

Definition: It is the type of traumatic dental injury that displaces the tooth deeper into the alveolar bony socket. It exhibits reduced mobility and gives a high metallic sound on percussion [5,28]. Severely intruded teeth frequently exhibit replacement resorption, marginal bone loss, and loss of vitality, with a higher prevalence of root resorption and pulp necrosis in mature teeth [3,28]. The probability of a deeply intruded tooth to erupt spontaneously is minimal. Thus, monitoring for spontaneous eruption is applicable only in very mild intrusions [30]. It is safe to say that orthodontic repositioning might cause various results and cannot be predicted based on the appearance or extent of the injury [28].

Orthodontic management: Orthodontic extrusion must be considered for moderate to severe intrusion (6-7 mm) cases [5,29]. If the intrusion is greater than 6-7 mm, immediate orthodontic repositioning and surgical repositioning are the obvious choices [29]. All severely and moderately intruded teeth with closed apices should be repositioned rapidly to allow access for extirpation of a non-vital pulp within three to four weeks after trauma to prevent inﬂammatory root surface resorption [5,3]. When a sectional fixed appliance is planned to realign intruded teeth, bracket positioning on the adjacent teeth should be passive, as its role is only to assist in correcting the position of traumatized teeth and not aligning the adjacent teeth. An observation period of 6-12 months before comprehensive orthodontic treatment is recommended for complete periodontal healing [5].

Avulsion

Definition: The tooth is entirely displaced out of the socket. Such teeth may have been replanted and splinted [5,28].

Orthodontic management: An observation period of 6-12 months before orthodontic treatment is recommended in order to ensure that periodontal healing is established and to prevent extra-inflammatory stimuli, which may further increase the risk of ankylosis [5,6,28]. Sometimes, the tooth can be repositioned but the hydraulic pressure of the blood or the oozing of blood in the socket prevents complete seating. For such cases, orthodontic repositioning may be considered as discussed for lateral luxation injuries [29].

Crown and crown/root fracture

A crown fracture is defined as a fracture that involves the enamel, dentine, and possibly the pulp. A crown-root fracture is a fracture that involves the enamel, dentin, and cementum and possibly the pulp (complicated or uncomplicated fractures) [5,28]. For patients with mature roots and crown trauma, tipping movements may result in a signiﬁcant risk of root resorption (28%) and pulp pathology (7%) when compared with orthodontic treatment of non-traumatized teeth [29].

Orthodontic Management

An observation period of three months is recommended before comprehensive orthodontic treatment for the establishment of a hard tissue barrier around the involved pulp [3,28]. In adjunctive orthodontic treatment, before extruding the tooth to bring the fracture margin supragingival for restoration, root canal treatment should be performed, and then rapid extrusion aiming to displace the fracture line coronally without moving the marginal bone. By doing so, the need for doing further marginal bone reshaping will be minimized [28]. The adverse effect of coronal migration of the gingiva and bone around the orthodontically extruded tooth can be prevented by performing fiberotomy that aims to section the supra-crestal gingival ﬁbers. This procedure should be performed at the beginning of orthodontic extrusion and repeated weekly or every two weeks, with simultaneous root planning [8]. When extruding a fractured tooth, it is ideal to use a stabilizing arch-wire (e.g., 0.018-inch stainless steel wire) to the adjacent teeth with a flexible ‘piggyback’ arch-wire (e.g., 0.012-inch NiTi) to extrude the fractured tooth with approximately 50 g force levels [8]. During extrusion, the bracket should be positioned very close to the cemento-enamel junction. An alternative option is to bond the wire directly on the surface of the injured tooth and including up to four anchor teeth to minimize adverse effects. The incisal edge of the affected tooth should be shortened up to 1 mm to allow for adequate eruption and to avoid occlusal interference [8]. Clinical and radiographic assessment has to be done at six months, one year, and two years after starting orthodontic treatment [28].

Root fracture

Root fractures are fractures that involve the roots of teeth in horizontal or oblique planes. Their clinical findings include bleeding from gingival sulcus, tenderness to percussion, mobility of coronal fragment, crown discoloration, and/or negative sensibility tests [5]. Orthodontic treatment of such teeth might cause separation of the segments and can result in a high prevalence of loss of vitality and pulp canal obliteration (PCO) [31]. The orthodontic management of teeth with root fractures depends on two factors: the fracture site and the healing type at the fracture site [28].

Cervical-Third Root Fracture

Orthodontic management: If the fracture happens without the separation of fragments and has a positive response to sensibility testing from the coronal portion, the tooth may need to be splinted for up to four months, and the fracture must be observed for at least two years prior to orthodontic tooth movement [3,6,31]. If the separation of the fragments has occurred, the displaced coronal segment must be repositioned prior to splinting; if this is not possible, the clinician may consider disposing the fragment and extruding the apical segment for restorative purposes [6].

Middle Third or Apical Third Root Fracture

Orthodontic management: An observation period of one to two years before comprehensive orthodontic treatment is recommended, or a shorter period, if the tooth was asymptomatic and healing was done by hard tissue (i.e., formation of dentin and cementum at the site of fracture), and then continuing to follow up the case through out the treatment [3,5,28,31]. Teeth with root fractures that healed with granulation tissue at the fracture site are not considered good candidates for orthodontic movement [28]. If healing by connective tissue has occurred, then the coronal fragment must be considered as a tooth with a short root, and orthodontic movement must not commence until successful root canal treatment and connective tissue healing has occurred [3,5]. The apical root portion will remain vital and will usually get separated from the coronal fragment when the coronal fragment is subjected to orthodontic movement [5]. The orthodontist should provide specific warnings regarding the expected compromise in the crown/root ratio and the expected mobility and possible tooth loss when orthodontic movement of such teeth is planned [5].

Endodontically treated teeth secondary to trauma

These are teeth that previously suffered from injuries that caused the pulp condition to become compromised and required endodontic treatment with gutta-percha [5]. When orthodontic tooth movement commences immediately after endodontic treatment following moderate-to-severe dental trauma, an additional inflammatory stimulus may prolong the destructive phase acting on the cementum, thereby increasing the risk of ankylosis. Endodontic treatment is considered successful one-year post-treatment with the absence of symptoms such as pain, swelling, sinus tract and hindered function, along with radiographic evidence of a sound periodontal ligament space around the root [6,3].

Orthodontic Management

The observation period before orthodontic treatment should be of one year to enable monitoring of healing and ankylosis, followed by radiographic monitoring six months after the start of orthodontic treatment. If there are signs of resorption, the patient should be informed and a further rest period of three months is needed before reassessment for further orthodontic movement [3,5,6]. If the endodontic treatment was required secondary to dental caries, orthodontic movement may commence immediately [3,6]. Teeth requiring root canal treatment, during or prior to orthodontic tooth movement, may benefit from being initially prepared and dressed with calcium hydroxide. It has been recommended that dressings should be replaced every three to six months until the end of orthodontic treatment, when final obturation with gutta-percha can be completed. It is thought that calcium hydroxide may halt root resorption, because its alkalinity is believed to impede osteoclastic and cementoclastic activities [6]. However, prolonged calcium hydroxide dressings (for longer than 30 days) can lead to a higher risk of cervical root fracture. Thus, if prolonged orthodontic tooth movement is planned, definitive obturation and a well-sealed coronal restoration should be placed as soon as possible, unless the calcium hydroxide is being used for apexification or disinfection [6].

Endodontic challenges

Pulp Canal Obliteration

PCO occurs more frequently in teeth with open apices that have suffered from severe luxation injuries and root fractures. Teeth with subluxation and crown fractures also may exhibit PCO, although with a lower frequency [2]. PCO is a sign of continued vitality and needs no treatment other than regular, close radiographic monitoring [5].

Orthodontic management: The risk of pulp necrosis after orthodontic movement of teeth with PCO should be carefully explained to the patient along with the difficulties that might be encountered in performing endodontic treatment, if needed, and an informed consent must be obtained [5]. Partial or complete exclusion of teeth with PCO from orthodontic forces is beneficial, when possible. Short-acting, light forces should generally be used (<70 g) [5].

Infection-Related Resorption

Orthodontic management: Whenever there is evidence of infection-related external resorption, root canal treatment should be initiated immediately. The canal should be medicated with calcium hydroxide for three weeks and replaced every three months until the radiolucencies of the resorptive lesions disappear. The final obturation of the root canal can be performed when bone repair is visible radiographically [2]. Orthodontic movement can only be initiated once the infection is controlled and the results are stable. Routine radiographic monitoring throughout the course of treatment is necessary for such teeth. The objectives of orthodontic treatment could be modified or limited at the beginning of treatment [5]. The exclusion of such teeth from orthodontic forces, whether partial or complete exclusion, is beneficial when possible [5].

Replacement Resorption (Ankylosed Teeth)

Orthodontic management: A multidisciplinary team is recommended for the management of such teeth as they may require advanced treatment modalities, such as tooth auto-transplantation or decoronation or forced luxation followed by orthodontic extrusion [5]. The objectives of orthodontic treatment could be modified or limited at the beginning of treatment with a distinct view of long-term alternative treatments in case the tooth has been lost [5]. Such teeth should be excluded from orthodontic forces or utilized for anchorage where possible.

Tooth Auto-transplantation

Auto-transplantation is a traditional method in dentistry to recreate a functional tooth substitution and may provide an often overlooked treatment option in hypodontia cases requiring orthodontic treatment [5,32]. This involves the extraction of the premolar with an immature root and its transplantation into a surgically prepared socket elsewhere in the mouth, placing it in infra-occlusion, and then orthodontically moving it over a period of approximately three to four months [6,29]. Early anticipation of this type of treatment must be done and it can be attempted only during certain windows of time with the developing teeth [29]. Most complications associated with auto-transplantation are ankylosis and root resorption [32].

Orthodontic management: A clear referral pathway has to be established to prevent unnecessary delays and unnecessary complications. Auto-transplantation is best performed when the premolar root is three-quarters formed [5]. Orthodontic tooth movement following auto-transplantation should start three to nine months after surgery, i.e., approximately eight weeks, once the periodontal ligament has healed and prior to complete alveolar healing [3,6]. A clear post-transplantation orthodontic treatment plan must be formulated [5]. The commencement of extrusion movement may be done earlier than bodily or rotational tooth movement [3].

Regenerative Endodontic/Revitalisation Technique

Orthodontic management: The objectives of orthodontic treatment could be modified or limited at the beginning of treatment. An observation period of minimum two years before orthodontic treatment is advised, until the final results are stable [5]. A multidisciplinary team is recommended for the management of such teeth as they may require advanced treatment modalities, such as tooth auto-transplantation or decoronation [5].

Apicected Teeth

Orthodontic management: The early application of orthodontic force has been proven to delay the healing process [6]. Periapical lesions should show good radiographic healing one year following apicectomy treatment before commencing orthodontic treatment [6].

Guidelines for the management of traumatized and endodontically treated teeth are summarized in Table 1 [6].

**Table 1:** Summary of orthodontic management guidelines for traumatized and endodontically treated teeth Reference studies [3,5,6]

Guidelines	
Immature traumatized teeth	Observe for evidence of continued root development in the radiographs. Clinical and radiographic evaluation must be carried out at six months, one year and two years after the trauma.
Minor damage to the periodontium	A three-month observation period is recommended to rule out inflammatory root resorption.
Moderate/severe damage to the periodontium	One year of observation period is needed to rule out ankylosis. Orthodontic tooth movement can only be started after periodontal healing has been completed that occurs at six months. If teeth are orthodontically moved between 6 and 12 months, a strong suspicion for ankylosis is observed, especially when the tooth movement is not as expected.
Crown and crown/root fractures without pulpal involvement	A three-month observation period is recommended to rule out inflammatory resorption.
Crown and crown/root fractures with pulpal involvement	Orthodontic movement can be commenced after vital pulp therapy and radiographic signs of a hard tissue barrier are evident (approximately three months).
Root fractures	A one- to two-year observation period is recommended, and a shorter period is recommended if asymptomatic. When healing is achieved by connective tissue, the coronal segment must be treated as a tooth with a short root and the tooth should not be moved until successful endodontic treatment and connective tissue healing of the coronal fragment have occurred.
Teeth requiring endodontic treatment secondary to caries	Immediate orthodontic movement is recommended in the absence of periapical pathosis. Definitive obturation is recommended with gutta-percha, rather than using calcium hydroxide in the root canal.
Teeth requiring endodontic treatment due to trauma	In mature teeth, following an initial dressing of calcium hydroxide, a definitive obturation with gutta-percha should be placed. This contradicts previous advice given by others. The observation period before orthodontic treatment should be of one year to enable monitoring of healing and ankylosis. Then routine radiographic monitoring is advised every six months.
Pulp canal obliteration	This is not an indication for endodontic treatment, as the tooth is still vital. Radiographic monitoring is advised. Light, short-acting forces are advised if necessary. Partial or complete exclusion of such teeth from orthodontic forces is beneficial, when possible.
Infection-related resorption	Orthodontic treatment is started only when infection is under control. A multi-disciplinary team is recommended.
Teeth requiring endodontic treatment due to inflammatory resorption	Radiographic evidence of healing should be awaited with an observation period of at least one year before the commencement of orthodontic tooth movement; tooth with signs of root resorption is considered more liable for further resorption during orthodontic treatment.
Replacement resorption	A multi-disciplinary team is recommended for the possible need of auto-transplantation or decoronation. Treatment objectives should be limited. Pulp and root health records at baseline and during treatment should be maintained. Forced luxation followed by orthodontic extrusion should be considered for alignment to the final position. The tooth should be left off arch-wire or utilized for anchorage.
Auto-transplanted teeth	Orthodontic treatment can be commenced after three to nine months after periodontal healing (approximately eight weeks) and prior to complete bone repair. The commencement of extrusion may be done earlier than rotational or bodily tooth movements. Ankylosis must be excluded when the tooth is not moving as expected.
Regenerative endodontic/revitalisation technique	Orthodontic treatment should be deferred until results are stable, with a minimum observation period of two years.
Apicected teeth	Periapical lesions should show good radiographic healing one year following apicectomy treatment before commencing orthodontic treatment.

## Conclusions

This review was put together in response to researches highlighting the need for further training and education in the orthodontic management of traumatized and endodontically treated teeth, as it is a challenge that frequently confronts orthodontists. It is imperative for orthodontists to be familiar with the techniques and aware of the appropriate timing to proceed with orthodontic tooth movement of traumatized as well as endodontically treated teeth, in order to avoid root resorptions and pulpal and periodontal consequences.
